# Supplementary material for: Ectopic Expression of Jatropha curcas TREHALOSE-6-PHOSPHATE PHOSPHATASE J Causes Late-Flowering and Heterostylous Phenotypes in Arabidopsis but not in Jatropha
Source: Int J Mol Sci. 2019 May 1;20(9):2165. doi: 10.3390/ijms20092165 (PMC6540179; doi:10.3390/ijms20092165)
Supplement: Supplementary file 1 [file ijms-20-02165-s001.pdf]

Supplementary Files

Ectopic expression of *Jatropha curcas* *TREHALOSE-6-PHOSPHATE PHOSPHATASE J* causes late-flowering and heterostylous phenotypes in *Arabidopsis* but not in *Jatropha*

Mei-Li Zhao[1,2], Jun Ni[1], Mao-Sheng Chen[1,*], Zeng-Fu Xu[1,*]

[1] CAS Key Laboratory of Tropical Plant Resources and Sustainable Use, Xishuangbanna Tropical Botanical Garden, The Innovative Academy of Seed Design, Chinese Academy of Sciences, Menglun, Mengla, Yunnan 666303, China

[2] College of Life Sciences, University of Chinese Academy of Sciences, Beijing 100049, China

zhaomeili@xtbg.ac.cn (M.-L. Z.); nijun@ipp.ac.cn (J. N.)

[*] Correspondence: chenms@xtbg.org.cn (M.-S. C.); zfxu@xtbg.ac.cn (Z.-F. X.)

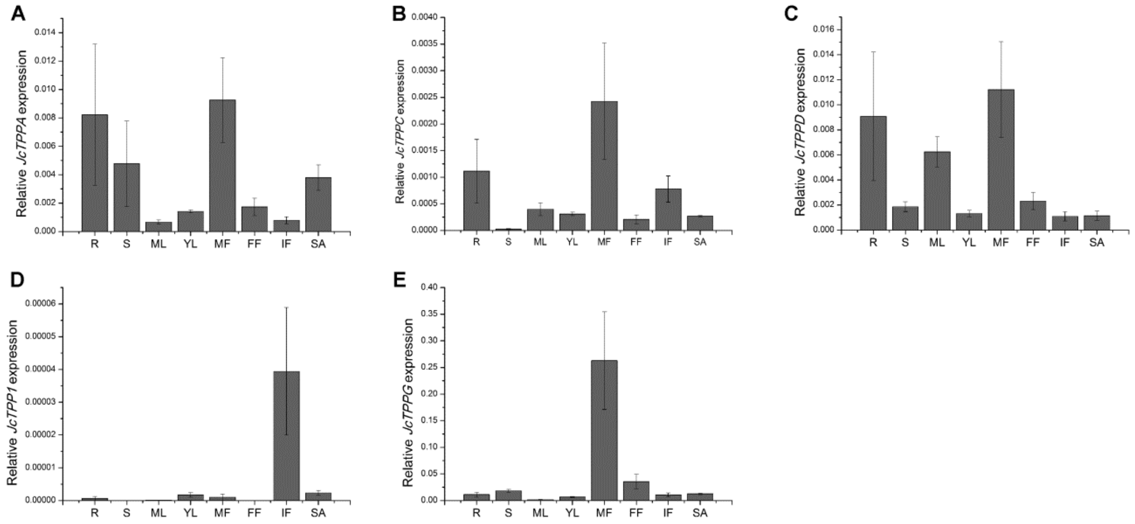

Figure S1. Relative expression levels of *JcTPPs* in various organs of adult *Jatropha*. Expression of *JcTPPA* (A), *JcTPPC* (B), *JcTPPD* (C), *JcTPP1* (D) and *JcTPPG* (E) in various organs of adult *Jatropha*. The qRT-PCR results were obtained from three independent biological replicates per sample. The levels of detected amplification were normalized using the amplified products of *JcGAPDH* genes as a reference. The error bars represent the standard error. R, roots; S stems; ML, mature leaves; YL, young leaves; FF, female flowers; MF, male flowers; IF, inflorescence buds, and SA, stem apices.

Table S1. GenBank accession numbers of the selected *TPP* genes in the phylogenetic analysis shown in Figure 1.

**Species**	**Protein**	**GenBank accession number**	**GenBank accession number of nucleotide sequences**
Jatropha	JcTPP1	XP 012083824.1	_
	JcTPPA	XP 020540837.1	_
	JcTPPC	XP 012067842.1	_
	JcTPPD	this paper	MK587442
	JcTPPG	this paper	MK587443
	JcTPPJ	this paper	MK587444
	JcTPS1	XP 012076095.1	_
Arabidopsis	AtTPS1	NP 001322932.1	_
	AtTPPA	NP 001332432.1	_
	AtTPPB	NP 177932.1	_
	AtTPPC	F4I1A6.1	_
	AtTPPD	NP 564464.1	_
	AtTPPE	Q67X99.1	_
	AtTPPF	Q9SU39.1	_
	AtTPPG	Q9SUW0.1	_
	AtTPPH	Q8GWG2.1	_
	AtTPPI	F4KFG5.1	_
	AtTPPJ	Q5HZ05.1	_
Escherichia coli	otsB	NP 416411.1	_

Table S2. Primers used in this study.

Primer name	Primer sequence	Utility
JcTPPA-fw	GTCTAATGCTATGCGTTCTGCTGTCA	qRT-PCR
JcTPPA-rev	CACCTTATCACGGCTTCTTCCACTAA	
JcTPPC-fw	ATTAGTGGTAGGAGCAGAGACAAGGT	
JcTPPC-rev	TGGTCTTGGTGGTGCCATAATGTC	
JcTPPD-fw	TAGTGAGATGCGAGAGGCTGTTAGA	
JcTPPD-rev	CCTTGTCTCTGCACCTTCCAGTAAC	
JcTPP1-fw	ACACCAATAGTTGATGATCCTGCTCG	
JcTPP1-rev	TTGTTGCGGCTCCTTCCACTTAC	
JcTPPG-fw	GTTCGCTCCAATGGATGGCTTGA	
JcTPPG-rev	AGTAGTCGGCAATGTCAGTCTCATCT	
JcTPPJ-fw	TGGGACAAAGGGAAGGCTCTTGA	
JcTPPJ-rev	TCATCCGTGCGATCATCTCCGATA	
JcTPPJ-fw	CGGGATCCGGAAATTAATTAGAGCCAAGATGAC	Amplication
JcTPPJ-rev	GCCGGAATTCTACTCTTTACATCCTTGGTTG	
